# Enhanced membrane binding of oncogenic G protein αqQ209L confers resistance to inhibitor YM-254890

**DOI:** 10.1016/j.jbc.2022.102538

**Published:** 2022-09-27

**Authors:** Clinita E. Randolph, Morgan B. Dwyer, Jenna L. Aumiller, Alethia J. Dixon, Asuka Inoue, Patrick Osei-Owusu, Philip B. Wedegaertner

**Affiliations:** 1 Department of Biochemistry and Molecular Biology, Sidney Kimmel Medical College, Thomas Jefferson University, Philadelphia, Pennsylvania, USA; 2 Department of Physiology and Biophysics, Case Western Reserve University School of Medicine, Cleveland, Ohio, USA; 3 Graduate School of Pharmaceutical Sciences, Tohoku University, Sendai, Japan

**Keywords:** heterotrimeric G protein, cell signaling, GTPase, plasma membrane, protein targeting, protein acylation, oncogene, BSA, bovine serum albumin, DAG, diaacylglycerol, DMSO, dimethyl sulfoxide, GPCR, G protein–coupled receptor, Ni-NTA, nickel nitrilotriacetic acid, PM, plasma membrane

## Abstract

Heterotrimeric G proteins couple activated G protein–coupled receptors (GPCRs) to intracellular signaling pathways. They can also function independently of GPCR activation upon acquiring mutations that prevent GTPase activity and result in constitutive signaling, as occurs with the αqQ209L mutation in uveal melanoma. YM-254890 (YM) can inhibit signaling by both GPCR-activated WT αq and GPCR-independent αqQ209L. Although YM inhibits WT αq by binding to αq-GDP and preventing GDP/GTP exchange, the mechanism of YM inhibition of cellular αqQ209L remains to be fully understood. Here, we show that YM promotes a subcellular redistribution of αqQ209L from the plasma membrane (PM) to the cytoplasm. To test if this loss of PM localization could contribute to the mechanism of inhibition of αqQ209L by YM, we developed and examined N-terminal mutants of αqQ209L, termed PM-restricted αqQ209L, in which the addition of membrane-binding motifs enhanced PM localization and prevented YM-promoted redistribution. Treatment of cells with YM failed to inhibit signaling by these PM-restricted αqQ209L. Additionally, pull-down experiments demonstrated that YM promotes similar conformational changes in both αqQ209L and PM-restricted αqQ209L, resulting in increased binding to βγ and decreased binding to regulator RGS2, and effectors p63RhoGEF-DH/PH and phospholipase C-β. GPCR-dependent signaling by PM-restricted WT αq is strongly inhibited by YM, demonstrating that resistance to YM inhibition by membrane-binding mutants is specific to constitutively active αqQ209L. Together, these results indicate that changes in membrane binding impact the ability of YM to inhibit αqQ209L and suggest that YM contributes to inhibition of αqQ209L by promoting its relocalization.

Heterotrimeric G proteins, comprised of an α, β, and γ subunit, act as molecular switches to regulate cell signaling pathways (1, 2, 3). Heterotrimeric G proteins couple to G protein–coupled receptors (GPCRs), which upon activation act as guanine-nucleotide exchange factors (GEFs) to induce conformational changes that promote GDP release from the α subunit in exchange for GTP (2, 3). Binding of GTP to the α subunit results in dissociation of the α subunit from the tightly associated βγ subunit and subsequent binding of α and βγ to effector proteins. α subunits are grouped into four families (αs, αi, αq, and α12/13) that mediate various pathways within the cell (4). The Gαq family, which is comprised of αq, α11, α14, and α15/16, classically activate phospholipase Cβ (PLCβ) (5, 6). Active PLCβ hydrolyzes phosphatidylinositol 4,5-bisphosphate resulting in production of inositol 1,4,5-trisphosphate and diacylglycerol (DAG) (7).

αq signaling is essential for physiological processes; however, activating mutations in α subunits can result in dysregulation of signaling and disease. αq and α11, which share 90% identity at the amino acid level and signal similarly, are mutated mutually exclusively in over 90% of uveal melanoma cases resulting in their constitutive activity (8, 9, 10). α subunits are comprised of a helical domain and a GTPase domain. The helical domain and GTPase domain form a crevice where the guanine nucleotide binds (11), and the GTPase domain contains three critical switch regions (Sw I, Sw II, and Sw III) that undergo conformational changes allowing for GTP binding. The switch regions are also important for intrinsic GTP hydrolysis activity, which is accelerated by GTPase-activating proteins (GAPs). αq/11 mutations in uveal melanoma most frequently involve a missense mutation at glutamine 209 to leucine or less commonly proline. The mutation at glutamine 209 located in Sw II greatly diminishes the ability of αq or α11 to hydrolyze GTP rendering them constitutively active (12). The implications of αq mutations in disease warrants an urgent need to better understand the biology of αq under physiological and disease conditions.

Two major signaling pathways that are activated by constitutively active αq to promote cell growth and proliferation are the mitogen-activated protein kinase (MAPK) pathway and the nuclear translocation of the transcription coactivators Yes-associated protein 1 (YAP) and paralog transcriptional coactivator with PDZ-binding motif (TAZ). MAPK pathway activation occurs *via* increased DAG production upon αq activation of the classical effector PLCβ. DAG then serves a dual role in membrane recruitment of the RasGEF RasGRP3 and protein kinase C isoforms δ/ε (PKCδ/ε), which phosphorylate and further activate RasGRP3 (13, 14). The resulting activation of Ras then stimulates the well-known MAPK cascade. On the other hand, activated αq initiates the YAP/TAZ pathway by directly binding to and activating the RhoGEF Trio (15). Activation of Rho then leads to focal adhesion kinase (FAK)–dependent disruption of the cytoplasmic retention of YAP/TAZ, thereby promoting translocation of YAP/TAZ into the nucleus (16). Both the MAPK pathway and translocation of YAP into the nucleus promote transcription of cell growth and proliferative genes, resulting in uveal melanoma progression (13, 14, 15, 16).

A number of studies show the promise of YM-254890 (YM) and FR900359 (FR) as inhibitors of WT and constitutively active αq/11 (17, 18, 19, 20, 21). YM and FR are naturally occurring cyclic depsipeptides isolated from *Chromobacterium* and *Ardisia crenata*, respectively. Both compounds, having highly similar structures (22), are thought to have similar mechanisms of action of preventing the release of GDP from αq and thereby preventing activation by inhibiting the exchange of GDP for GTP (19, 21, 23). Although it is clear that FR/YM can effectively inhibit constitutive αqQ209L signaling, αqQ209L is thought to exist predominantly in the GTP-bound form due to the lack of GTP hydrolysis activity, presenting a paradox as to how FR/YM can trap αqQ209L in the GDP-bound form (24). Thus, the mechanisms of how constitutively active αqQ209L is regulated in cells and how FR/YM inhibit αqQ209L remain to be fully understood.

Importantly, membrane localization is critical for signaling by both WT and constitutively active αq. αq gains affinity for the plasma membrane (PM) through interaction with βγ (25, 26), palmitoylation at cysteines 9 and 10 (27), and an N-terminal polybasic motif (28). PM localization is particularly important for interaction with the GPCR and effector proteins such as PLCβ. Furthermore, mutational disruption of these membrane-targeting mechanisms of WT and constitutively active αq results in cytoplasmic localization and attenuation of signaling, demonstrating that PM localization is critical for αq signaling (25, 26, 27, 28, 29). Moreover, α subunits can traffic reversibly between the PM and intracellular organelles, highlighting the importance of subcellular localization for G protein signaling function (29, 30). Understanding further how localization regulates signaling by constitutively active αq is critical for gaining new insight into ways to inhibit dysregulated αq signaling.

In our studies, we provide evidence that YM treatment results in the redistribution of αqQ209L from the PM to the cytoplasm. Additionally, we generated PM-restricted αqQ209L mutants that displayed resistance to inhibition of signaling by YM. These studies suggest the importance of YM-induced translocation of αqQ209L into the cytoplasm as an additional way in which YM inhibits constitutively active αq.

## Results

### YM promotes the redistribution of αqQ209L from the PM to cytoplasm

**Figure 1:**
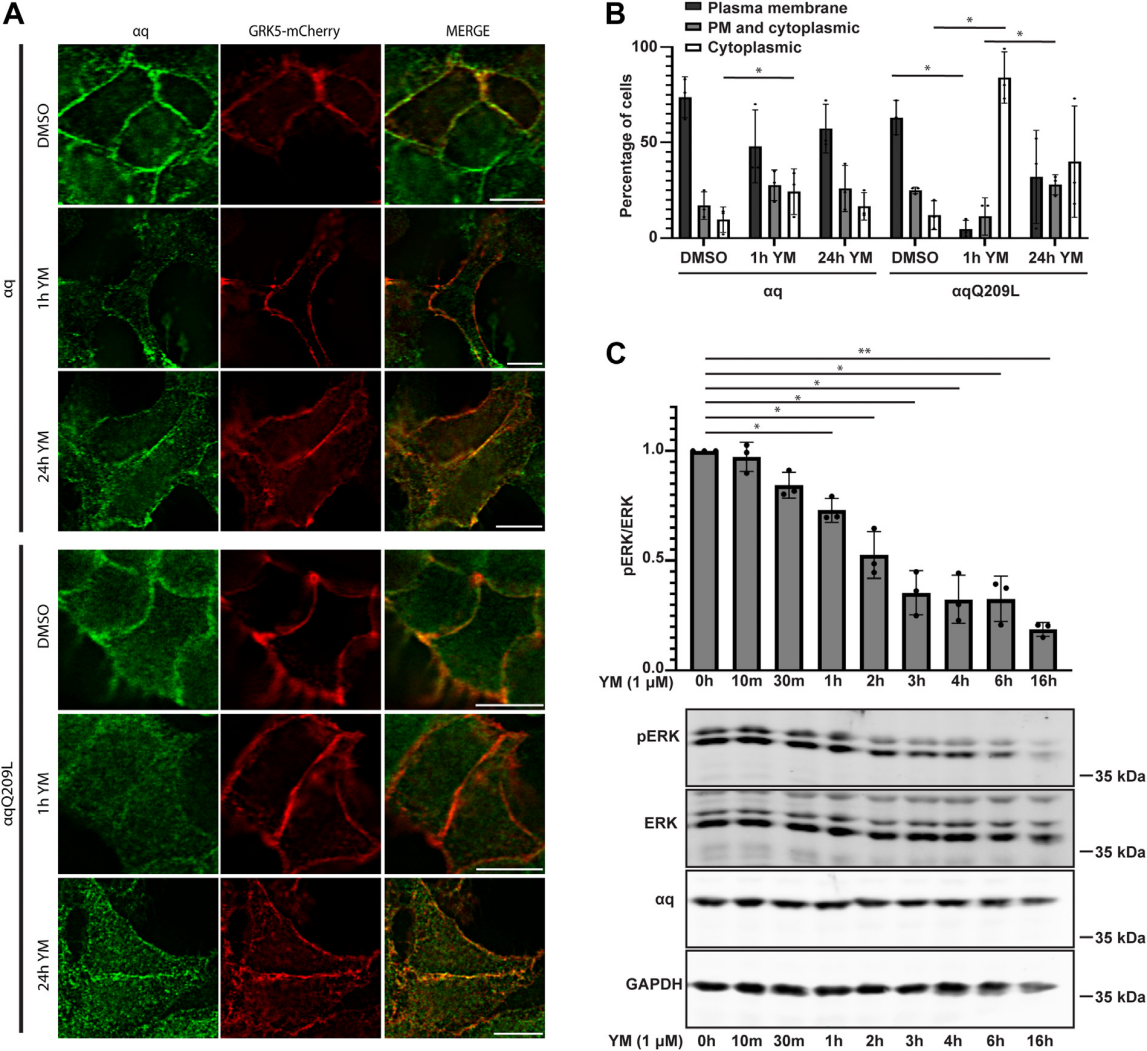
**YM promotes dissociation of αqQ209L from the PM.***A*, Flp-In HEK 293 cells were transfected with GRK5-mCherry and treated with tetracycline to induce expression of αq and αqQ209L. The tetracycline-induced αq- and αqQ209L-Flp-In HEK 293 cells were treated with DMSO or 1 μM YM for 1 h or overnight. αq, αqQ209L, and GRK5 were visualized by immunofluorescence microscopy, as described under Experimental procedures. The scale bars represent 10 μM. *B*, the localization of αq- or αqQ209L in 100 tetracycline-induced Flp-In HEK 293 cells in each of n = 3 experiments were scored as either PM localized with little to no observable staining in the cytoplasm, PM, and cytoplasmic localization in which individual cells displayed varying degrees of a partial PM stain and observable cytoplasmic localization of αq or cytoplasmic in which αq was distributed throughout the cytoplasm but had no observable PM localization. Statistical significance comparing experimental conditions was determined as described under Experimental procedures. Results are shown as mean ± SD (n = 3; experiments ∗*p* <0.05, two-way ANOVA, Fisher’s Least Significant Differences test). *C*, expression of αqQ209L was tetracycline induced in Flp-In HEK 293 cells, and cells were treated with 1 μM YM for the indicated times. Cell lysates were immunoblotted using antibodies for the indicated proteins. pERK and ERK signal intensities were quantified and normalized to pERK/ERK signal intensity in DMSO treatment. Results are shown as mean ± SD (n = 3; ∗*p* <0.05; ∗∗*p* <0.01, one-way ANOVA, Dunnett’s multiple comparisons test). DMSO, dimethyl sulfoxide; PM, plasma membrane.

While studying the effect of YM-254890 (YM) on αq-dependent signaling, we observed surprisingly that YM promoted a change in localization of αqQ209L and to a lesser extent αq WT. Immunofluorescence microscopy of HEK 293 cells stably expressing tetracycline-inducible αqQ209L showed that treatment of cells with 1 μM YM for 1 h promoted a redistribution of αqQ209L from a PM localization to an intracellular localization (Fig. 1*A*). Scoring αqQ209L localization in individual cells as PM localized, PM and cytoplasmic, or cytoplasmic showed that in dimethyl sulfoxide (DMSO) vehicle-treated cells, αqQ209L displayed a predominant PM localization in 63% of cells, colocalizing at the PM with GRK5, a protein shown to strongly localize at the PM when expressed in HEK 293 cells (31), and a distribution to both the PM and cytoplasm in 25% of cells (Fig. 1, *A* and *B*). However, after 1 h of YM treatment, only 5% of cells showed strong PM localization of αqQ209L; instead, αqQ209L was not detected at the PM and was localized in the cytoplasm in 84% of cells. Redistribution of αqQ209L was also observed after 24 h of YM treatment, but more PM localization remained compared to 1 h YM treatment (Fig. 1, *A* and *B*). Although αqQ209L displays a decrease in PM localization after YM treatment, GRK5 remains strongly localized at the PM, indicating that the observed redistribution of αqQ209L is not simply due to a general disruption in PM localization of peripheral membrane-bound proteins. In HEK 293 cells stably expressing tetracycline-inducible αq WT, a small but consistent shift-off of the PM for αq WT was observed after YM treatment (Fig. 1, *A* and *B*), but only αqQ209L displayed a dramatic redistribution. Translocation of αqQ209L in response to YM was also observed in HeLa cells (Fig. S1).

To begin to address whether the observed YM-promoted redistribution of αqQ209L is related to the ability of YM to inhibit constitutive signaling by αqQ209L, we examined a time course of YM inhibition. αqQ209L activates the MAPK pathway through its canonical signaling pathway mediated by PLCβ. Immunoblotting of cell lysates with a phospho-ERK (pERK)–specific antibody was used as a read out of MAPK signaling. In cells expressing αqQ209L, a significant decrease in pERK levels was detected after 1 h of YM treatment. pERK continued to decrease at 2 to 3 h and remained inhibited up to 16 h after YM treatment (Fig. 1*C*). These experiments together show that the YM-promoted redistribution of αqQ209L appears to precede YM-induced inhibition of signaling, raising the question of whether YM-promoted loss of PM localization of αqQ209L plays a role in the ability of YM to inhibit constitutive signaling by αqQ209L.

### YM does not disrupt the PM localization or signaling of Src-αqQ209L or Lyn-αqQ209L

**Figure 2:**
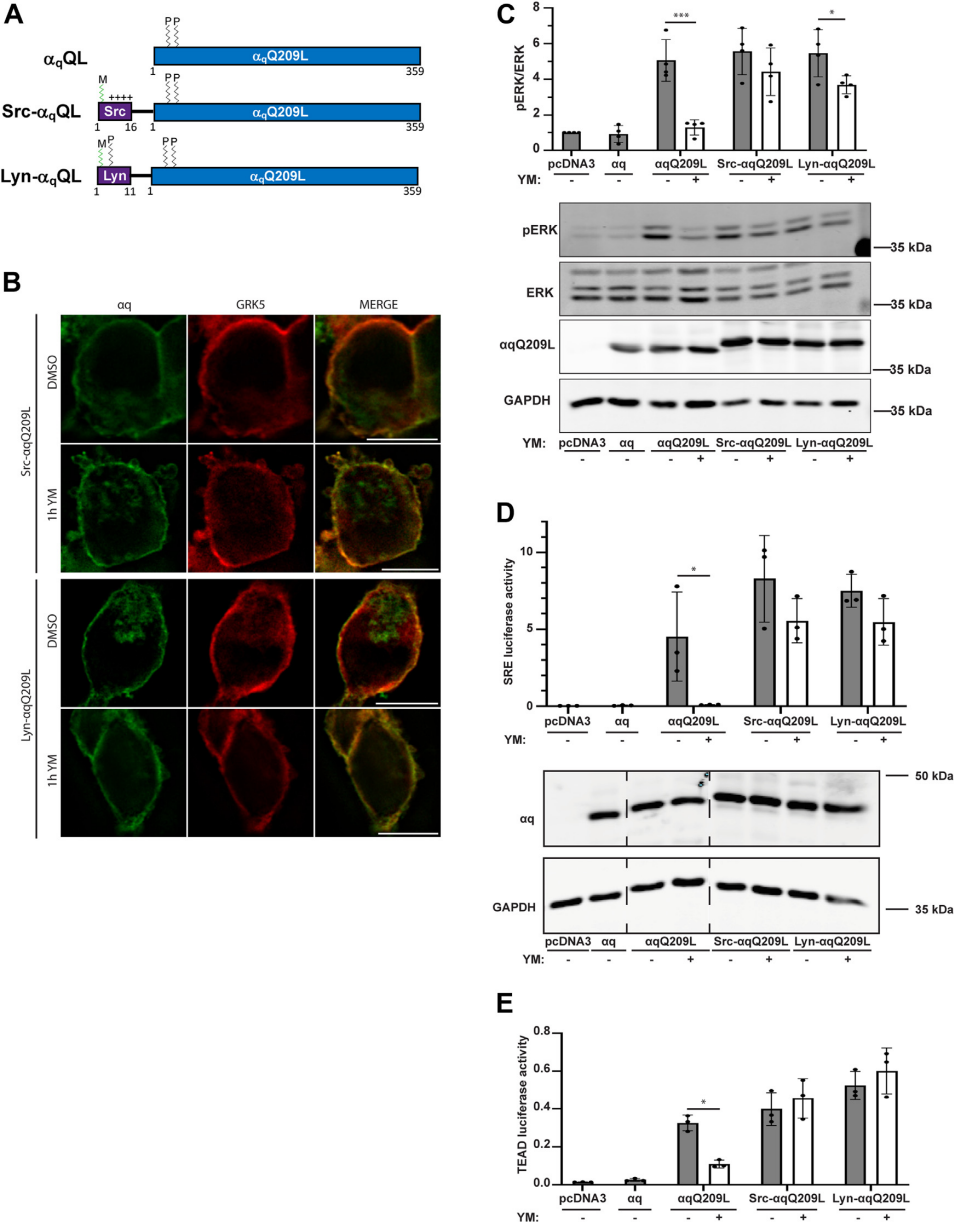
**Src- and Lyn-αqQ209L remain at the PM upon YM treatment and are resistant to inhibition by YM.***A*, schematic of αqQ209L, Src-αqQ209L, and Lyn-αqQ209L, indicating sites of palmitoylation (P), myristoylation (M), and a polybasic motif (++++). *B*, Src-αqQ209L or Lyn-αqQ209L was cotransfected with GRK5-mCherry in HEK 293 q/11 K/O cells and cells were treated with DMSO or 1 μM YM for 1 h. Src-αqQ209L, Lyn-αqQ209L, and GRK5-mCherry were visualized by immunofluorescence microscopy. The scale bars represent 10 μM. *C*, HEK 293 q/11 K/O cells were transfected with αq-pcDNA3, αqQ209L-pcDNA3, Src-αqQ209L-pcDNA3, Lyn-αqQ209L-pcDNA3, or pcDNA3 vector alone and treated with DMSO or 1 μM YM overnight. Cell lysates were immunoblotted using antibodies for the indicated proteins. pERK and ERK signal intensities were quantified and normalized to pERK/ERK signal intensity in DMSO treatment in pcDNA3-transfected cells. *D* and *E*, HEK 293 q/11 K/O cells were transfected with αq-pcDNA3, αqQ209L-pcDNA3, Src-αqQ209L-pcDNA3, Lyn-αqQ209L-pcDNA3, or pcDNA3 alone, along with *renilla* luciferase and either pSRE luciferase (*D*) or 8xGTIIC (TEAD) luciferase (*E*). Cell lysates were prepared, and luciferase assays were performed and quantitated as described under Experimental procedures. Cell lysates were immunoblotted using antibodies for the indicated proteins. In (*C*–*E*) results are shown as mean ± SD (n = 4 in *C*, n = 3 in *D* and *E*; ∗*p* <0.05; ∗∗*p* <0.01; ∗∗∗*p* <0.001, two-way ANOVA, Šidák’s multiple comparisons test). DMSO, dimethyl sulfoxide.

If redistribution from the PM plays a role in YM inhibition of αqQ209L signaling, we reasoned that a mutant of αqQ209L that remains at the PM upon YM treatment would continue to signal in the presence of YM. To test this idea, we initially constructed mutants of αqQ209L in which additional PM-targeting motifs were added to the N terminus to augment the ability of reversible palmitoylation at cysteines 9 and 10 to maintain PM localization. Amino acids 1 to 16 of Src or amino acids 1 to 11 of Lyn were fused to the N terminus of αqQ209L to introduce a myristoylation plus polybasic motif and a myristoylation plus palmitoylation motif, respectively (Fig. 2*A*). The addition of a similar N-terminal sequence of Src has been shown to strongly localize αs at the PM and prevent activation-induced subcellular redistribution of αs (32). Immunofluorescence microscopy showed that Src-αqQ209L or Lyn-αqQ209L displayed strong PM localization and colocalization with GRK5, when expressed in HEK 293 q/11 KO cells in which αq and α11 were knocked out using CRISPR (Fig. 2*B*). Importantly, Src-αqQ209L and Lyn-αqQ209L remained at the PM after YM treatment (Fig. 2*B*), thus demonstrating that the addition of the Src or Lyn motif is sufficient to prevent YM-promoted redistribution of αqQ209L.

To determine if these PM-restricted αqQ209L mutants are sensitive to inhibition by YM, we assayed pERK levels −/+ YM treatment by immunoblot analysis. Expression of αqQ209L, Src-αqQ209L, or Lyn-αqQ209L in HEK 293 q/11 KO cells resulted in similar constitutive activation of pERK, approximately 5-fold higher than in vector transfected or αq WT expressing cells (Fig. 2*C*). While YM treatment abolished αqQ209L activation of pERK, Src-αqQ209L- and Lyn-αqQ209L-dependent activation of pERK was strongly resistant to inhibition by YM. These results provide the first evidence that signaling by PM-restricted forms of αqQ209L is not effectively inhibited by YM.

To examine further this insensitivity to YM of PM-restricted αqQ209L, we utilized two transcriptional reporter assays. The serum response element (SRE) luciferase reporter assay detects αq-dependent signaling *via* both MAPK- and Rho-dependent pathways, and the TEAD luciferase reporter assay detects αq-dependent signaling *via* Rho-dependent translocation into the nucleus of the transcription coactivator YAP. Consistent with the pERK assay (Fig. 2*C*), expression of αqQ209L, Src-αqQ209L, or Lyn-αqQ209L in HEK 293 q/11 KO cells resulted in similar constitutive activation of SRE-luciferase and TEAD-luciferase signals (Fig. 2, *D* and *E*). However, and again consistent with the pERK assays, YM treatment resulted in little or no inhibition of signaling by Src-αqQ209L or Lyn-αqQ209L, even though αqQ209L signaling was abolished after YM treatment. Taken together, these results demonstrate that the addition of N-terminal PM-targeting motifs to αqQ209L does not affect constitutive signaling but does confer resistance to YM.

### Myristoylated αqAG-Q209L remains localized to the PM and is resistant to YM

**Figure 3:**
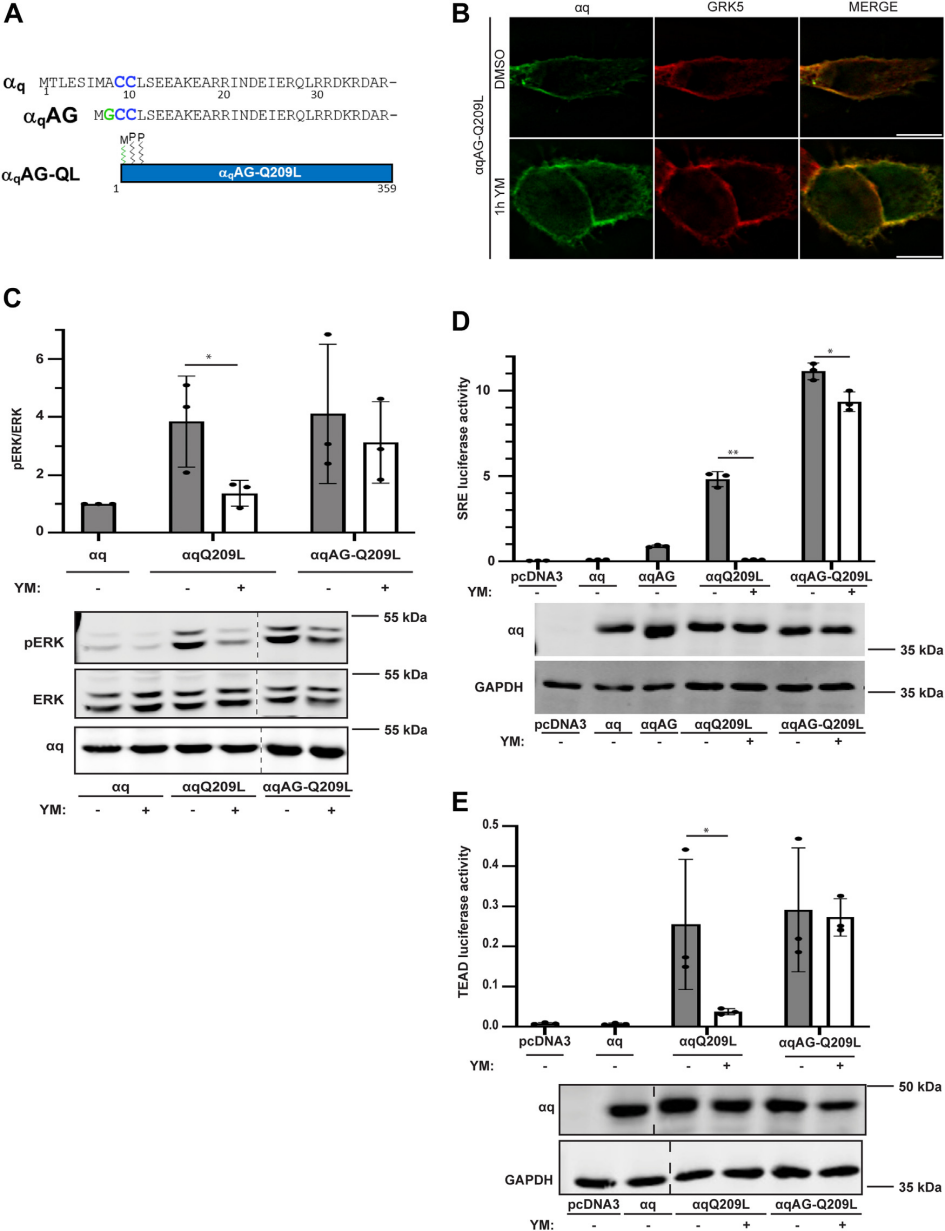
**αq-AGQ209 L remains at the PM upon YM treatment and is resistant to YM inhibition.***A*, schematic of αq, αqAG, and αqAGQ209L. *B*, αqAG-Q209L was cotransfected with GRK5-mCherry in HEK 293 q/11 K/O cells, and cells were treated with DMSO or 1 μM YM for 1 h. αqAG-Q209L and GRK5-mCherry were visualized by immunofluorescence microscopy. The scale bars represent 10 μM. *C*, HEK 293 q/11 K/O cells were transfected with αq-pcDNA3, αqQ209L-pcDNA3, αqAG-Q209L, or pcDNA3 vector alone and treated with DMSO or 1 μM YM overnight. Cell lysates were immunoblotted using antibodies for the indicated proteins. pERK and ERK signal intensities were quantified and normalized to pERK/ERK signal intensity in DMSO treatment in αq-transfected cells. *D* and *E*, HEK 293 q/11 K/O cells were transfected with αq-pcDNA3, αqAG-pcDNA3, αqQ209L-pcDNA3, αqAG-Q209L-pcDNA3, or pcDNA3 alone, along with *renilla* luciferase and either pSRE luciferase (*D*) or 8xGTIIC (TEAD) luciferase (*E*). Cell lysates were prepared, and luciferase assays were performed and quantitated as described under Experimental procedures. Cell lysates were immunoblotted using antibodies for the indicated proteins. In (*C*–*E*) results are shown as mean ± SD (n = 3: ∗*p* <0.05; ∗∗*p* <0.01, two-way ANOVA, Šidák’s multiple comparisons test). DMSO, dimethyl sulfoxide; PM, plasma membrane.

To further determine if signaling by PM-restricted αqQ209L fails to be inhibited by YM, we took advantage of a previously described N-terminal, membrane-binding mutant of αq. In this AG mutant, the first methionine codon is mutated so that αq utilizes the methionine codon at position 7 as the initiating ATG, and the alanine at position 8, now position 2 in the AG mutant, is changed to a glycine codon to introduce a new site for myristoylation (25). Thus, the αqAG-Q209L mutant is singly myristoylated and dually palmitoylated, in contrast to only dual palmitoylation for αqQ209L, thereby providing an additional lipid modification to increase PM localization (Fig. 3*A*). Consequently, immunofluorescence microscopy showed that αqAG-Q209L localized at the PM, colocalizing with GRK5 (Fig. 3*B*), and αqAG-Q209L remained at the PM after YM treatment (Fig. 3*B*). Thus, the simple introduction of a site for N-terminal myristoylation is sufficient to prevent YM-promoted redistribution of αqAG-Q209L, similar to that observed for the more complex Src-αqQ209L or Lyn-αqQ209L mutants (Fig. 2, *A* and *B*).

To determine if αqAG-Q209L signaling, like Src-αqQ209L and Lyn-αqQ209L signaling, was also insensitive to YM inhibition, we first examined pERK levels stimulated by αqAG-Q209L and the effect of YM treatment. Expression of αqAG-Q209L in HEK 293 q/11 KO cells resulted in strong constitutive activation of the MAPK pathway, as revealed by pERK immunoblotting, similar to αqQ209L (Fig. 3*C*). However, the constitutive pERK activation by αqAG-Q209L was resistant to YM treatment. Upon YM treatment, αqQ209L signaling to pERK was significantly reduced but αqAG-Q209L retained its pERK signaling. We also used SRE and TEAD luciferase reporter assays to further confirm αqAG-Q209L’s lack of inhibition of by YM (Fig. 3, *D* and *E*). Expression of either αqQ209L or αqAG-Q209L in HEK 293 q/11 KO cells resulted in strong constitutive activation in both luciferase transcriptional reporter assays. However, overnight YM treatment failed to inhibit SRE-dependent and TEAD-dependent luciferase activity in cells expressing αqAG-Q209L, even though YM treatment completely blocked signaling by αqQ209L. Collectively, our results demonstrate that enhanced membrane binding, displayed by three different PM-restricted αqQ209L mutants, prevents relocalization and inhibition by YM, supporting the idea that YM-induced subcellular redistribution of αqQ209L is important for inhibition of signaling by YM.

### YM insensitivity of PM-restricted αqAG-Q209L is not due to expression level or YM concentration

**Figure 4:**
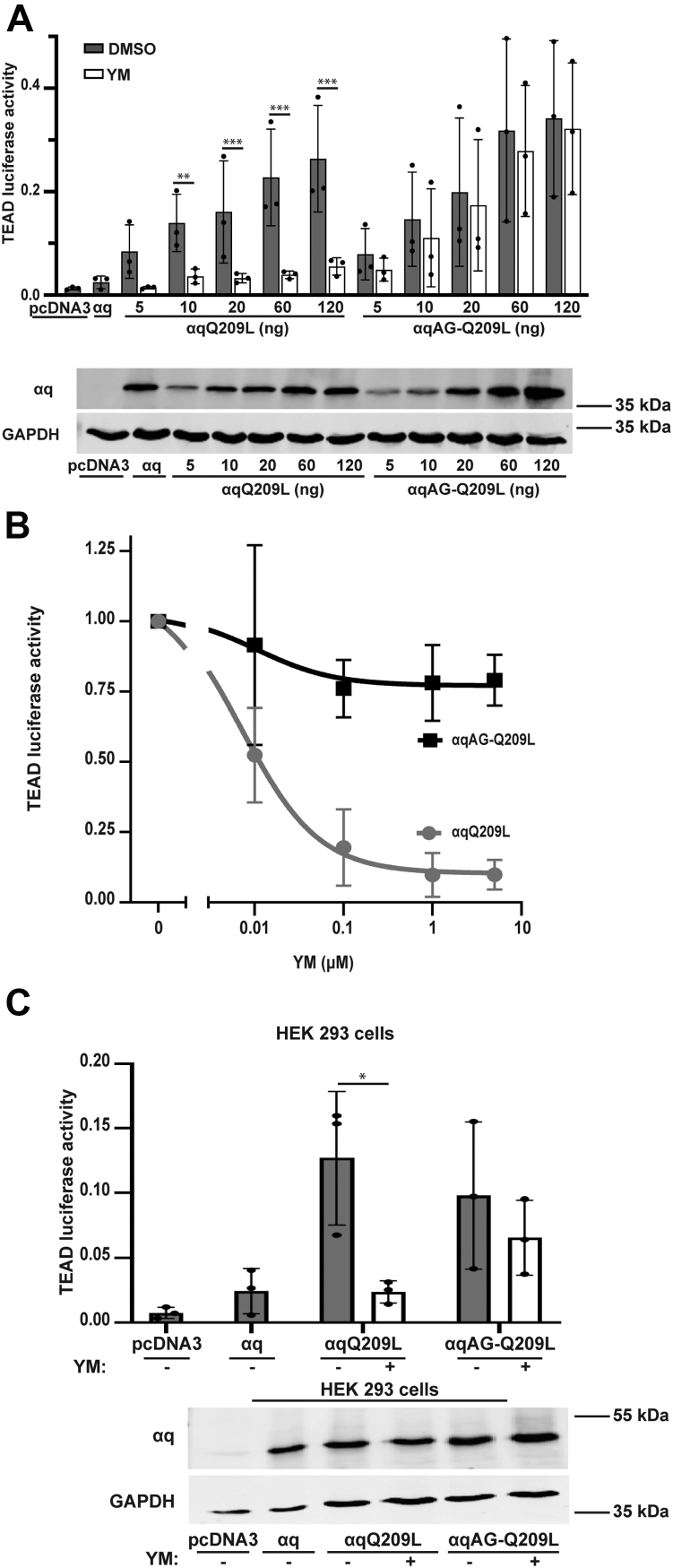
**The resistance of PM-restricted αqQ209L mutants to YM occurs independently of protein expression level, YM concentration, or presence of endogenous αq/11.***A*, HEK 293 q/11 K/O cells were transfected with *renilla* luciferase, 8xGTIIC luciferase, and increasing amounts of αqQ209L or αqAGQ209L, as indicated. Cells were treated with 1 μM YM overnight. Cell lysates were prepared, and luciferase assays were performed and quantitated as described. Cell lysates were also immunoblotted using antibodies for the indicated proteins. *B*, HEK 293 q/11 K/O cells were transfected with *renilla* luciferase, 8xGTIIC luciferase, and αqQ209L or αqAGQ209L. Cells were treated with increasing concentrations of YM overnight. Lysates were prepared, and luciferase assays were performed and quantitated. *C*, αqQ209L or αqAG-Q209L were cotransfected with *renilla* luciferase and 8xGTIIC luciferase in HEK 293 cells then treated with DMSO or YM overnight. Lysates were prepared, and luciferase assays were performed and quantitated. Cell lysates were also immunoblotted using antibodies for the indicated proteins. In (*A* and *C*) results are shown as mean ± SD (n = 3; ∗*p* <0.05; ∗∗*p* <0.01; ∗∗∗*p* <0.001, two-way ANOVA, Šidák’s multiple comparisons test). DMSO, dimethyl sulfoxide.

To address concerns that the aforementioned demonstrations of resistance to YM are artefacts of high levels of αqAG-Q209L expression or inappropriate concentrations of YM, we performed key control experiments. First, we assessed TEAD-dependent transcription using decreasing amounts of expression of αqQ209L and αqAG-Q209L. αqQ209L displayed a strong and significant decrease in TEAD luciferase activity upon YM treatment at both high and low expression levels (Fig. 4*A*). However, αqAGQ209L signaling remained resistant to YM at all expression levels, indicating that lack of inhibition by YM observed for this PM-restricted mutant was not due to high levels of expression. Second, we analyzed YM concentration curve. Using concentrations from 10 nM to 5 μM, we observed the expected YM concentration-dependent inhibition of αqQ209L signaling with an approximate IC50 of 9 nM, as measured here by TEAD luciferase activity. However, αqAG-Q209L signaling remained resistant to YM inhibition at all concentrations, including 5 μM YM (Fig. 4*B*). Additionally, we performed TEAD luciferase assays in HEK 293 cells, rather than the HEK 293 q/11 KO cells that lack endogenous αq and α11 (Fig. 4*C*). Signaling by expressed αqAG-Q209L in HEK 293 cells remained resistant to YM inhibition, indicating that this failure to be inhibited by YM occurs for PM-restricted αqAG-Q209L regardless of the presence or absence of endogenous WT αq and α11. Taken together, these results demonstrate that insensitivity to YM of PM-restricted αqAGQ209L is maintained at various expression levels of αqAG-Q209L, various concentrations of YM, and in HEK 293 parental cells.

### YM affects αqQ209L and PM-restricted αqAG-Q209L similarly in regulating binding to regulators and effectors

**Figure 5:**
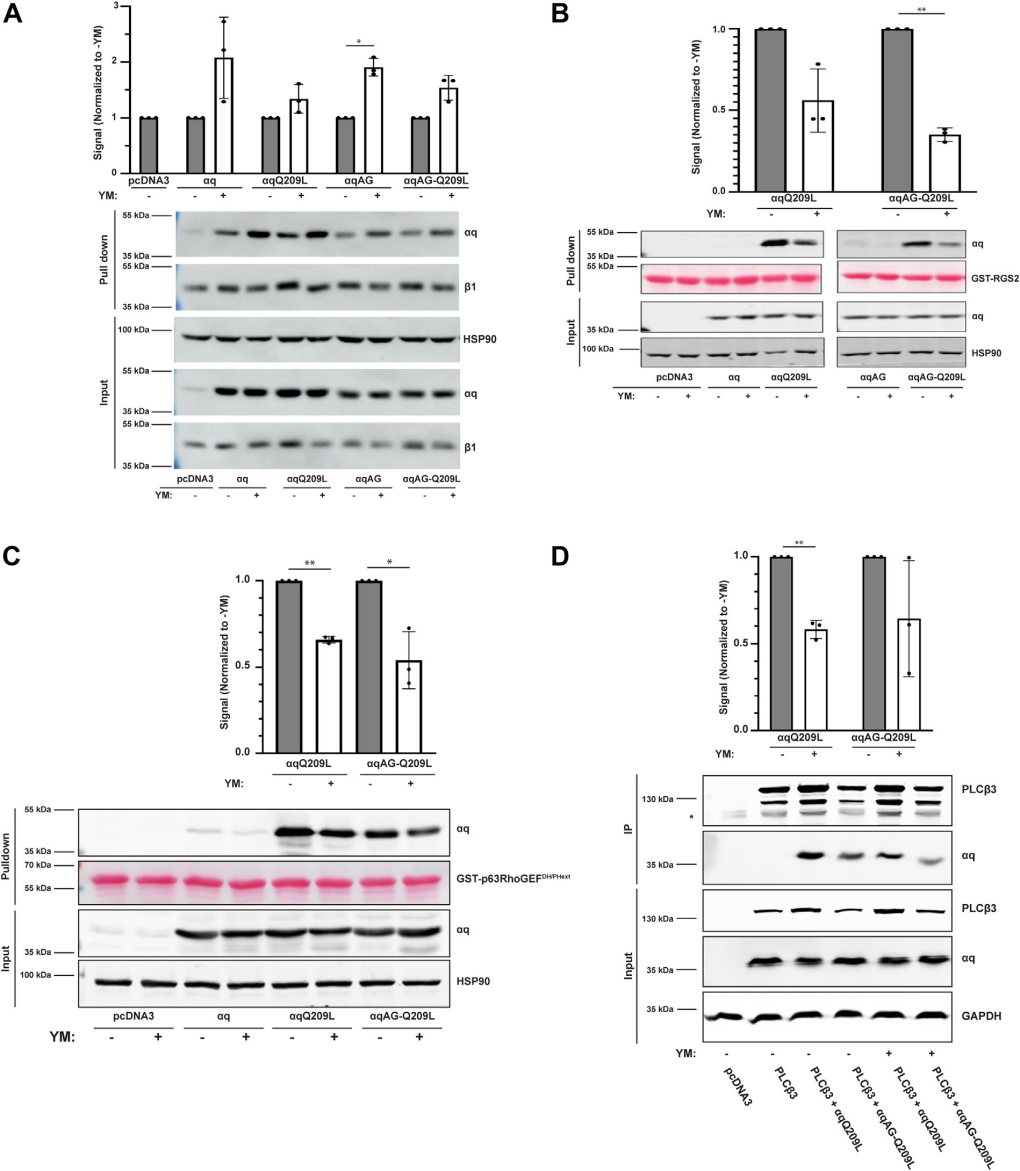
**PM-restricted αqQ209L displays YM-induced conformational changes.***A*, HEK 293 cells stably expressing His-Myc-β1γ2 were transfected with αq-pcDNA3, αqAG-pcDNA3, αqQ209L-pcDNA3, αqAG-Q209L-pcDNA3, or pcDNA3 vector alone then treated with 1 μM YM overnight. A βγ pull-down assay was done after the overnight YM treatment. Pull-down and whole cell lysates were immunoblotted using antibodies for the indicated proteins. αq signal intensities in the pull down were quantified and normalized to their respective signal intensities in untreated (-YM) cells to quantify the increase in association with β1γ2 after YM treatment. *B*, HEK 293 q/11 K/O cells were transfected with αq-pcDNA3, αqAG-pcDNA3, αqQ209L-pcDNA3, αqAG-Q209L-pcDNA3, or pcDNA3 vector alone then treated with 1 μM YM overnight. A GST-RGS2 pull-down assay was done after the overnight YM treatment. Pull-down and whole cell lysates were immunoblotted using antibodies for the indicated proteins, and GST-RGS2 in the pull down was visualized with Ponceau S staining. αqQ209L and αqAG-Q209L signal intensities in the pulldown were quantified and normalized to their respective signal intensities in untreated cells to quantify the decrease in association with RGS2 after YM treatment. *C*, HEK 293 q/11 K/O cells were transfected and treated with 1 μM YM as in *B*, and a GST-p63RhoGEF-DH/PHext pull-down assay was performed. Proteins were visualized and quantitation performed as in (*B*). *D*, HEK 293 q/11 K/O cells were transfected with αqQ209L-pcDNA3, αqAG-Q209L-pcDNA3, or pcDNA3 together with pcDNA3.1-FLAG-PLCβ-3 where indicated. FLAG-PLCβ-3 was immunoprecipitated from cell lysates as described under Experimental Procedures. Immunoprecipitates and cell lysates were immunoblotted using antibodies for the indicated proteins. The asterisk in the PLCβ-3 IP panel indicates a nonspecific band. Immunoprecipitated αqQ209L and αqAG-Q209L signal intensities were divided by the corresponding PLCβ-3 signal and then normalized to the respective signal intensities in the untreated sample. Results are shown as mean ± SD (n = 3; ∗*p* < 0.05, ∗∗*p* < 0.01, Student’s *t* test). PM, plasma membrane.

We next tested whether YM was binding to the PM-restricted mutants by assaying the ability of added YM to affect the interaction of αqAG-Q209L with several binding partners. Previous work showed that addition of YM to cells expressing αqQ209L promoted increased interaction with Gβγ and decreased interaction with regulator of G protein signaling 2 (RGS2), consistent with a proposed mechanism of YM binding to αqQ209L and shifting it into an inactive form (19). Here, we expressed αqQ209L and αqAG-Q209L, as well as the WT forms of αq and αqAG, in HEK 293 cells stably expressing poly-His-tagged β1. Previous work has demonstrated that Gβγ associates with αqQ209L similarly to its association with WT αq; the mechanism of such strong interaction of αqQ209L with Gβγ remains to be fully understood (26, 33). To observe the association of expressed αq subunits with Gβγ, we used nickel nitrilotriacetic acid (Ni-NTA) magnetic agarose beads to pull down poly-His-β1 from cell lysates and then immunoblotted for αq. These βγ pull-down assays showed that YM treatment promoted a similar increased interaction of both αqQ209L and αqAGQ209L with Gβγ upon YM treatment (Fig. 5*A*). Additionally, both WT αq and αqAG showed an expected increase in association with Gβγ upon YM treatment.

We also interrogated the ability of αqAG-Q209L to interact with RGS2 and the effect of YM. RGS2 binds to GTP-bound forms of αq and acts as a GTPase-activating protein (GAP) to stimulate the hydrolysis of GTP. Although the Q209L mutation greatly reduces αq’s intrinsic and RGS2-stimulated GTPase activity, αqQ209L is still able to associate with RGS2 (19). However, GDP-bound αq displays a poor affinity for RGS2, and therefore, reduced association of Q209L mutants with RGS2 in response to YM treatment would be consistent with YM binding and inducing an inactive conformation. To test this, we performed a GST-RGS2 pull-down assay, in which purified GST-RGS2, immobilized on GSH sepharose beads, was incubated with cell lysates containing the expressed αq subunits (Fig. 5*B*). The pull-down results showed that both αqQ209L and αqAG-Q209L displayed a significant and similar reduction in association with RGS2 after treating the cells with YM.

Similarly, binding to key effector proteins was examined. GST pull downs were performed with the extended PH/DH domain of p63RhoGEF, a direct effector of αq that stimulates activation of RhoA and downstream signaling (34). In addition, coimmunoprecipitation experiments were performed after coexpression of PLCβ-3 with αqQ209L or αqAG-Q209L (35). In both assays, αqQ209L and αqAG-Q209L were coisolated with the protein domain or effector protein at a similar level (Fig. 5, *C* and *D*). Consistent with the GST-RGS2 pull downs (Fig. 5*B*), treatment with YM reduced the level of αqQ209L or αqAG-Q209L that interacted with GST-p63RhoGEF-DH/PHext or PLCβ-3. Pull-down and immunoprecipitation experiments provide an important approach for monitoring changes in protein–protein interactions in response to YM; however, we note that protein–protein interactions detected in a cell lysate may not fully reflect the situation in the intact cell. Collectively, these results indicate that although YM fails to inhibit signaling mediated by PM-restricted αqAG-Q209L, YM is able to bind to and promote an inactive conformation of αqAG-Q209L.

### Signaling by GPCR-activated αqAG is sensitive to YM

**Figure 6:**
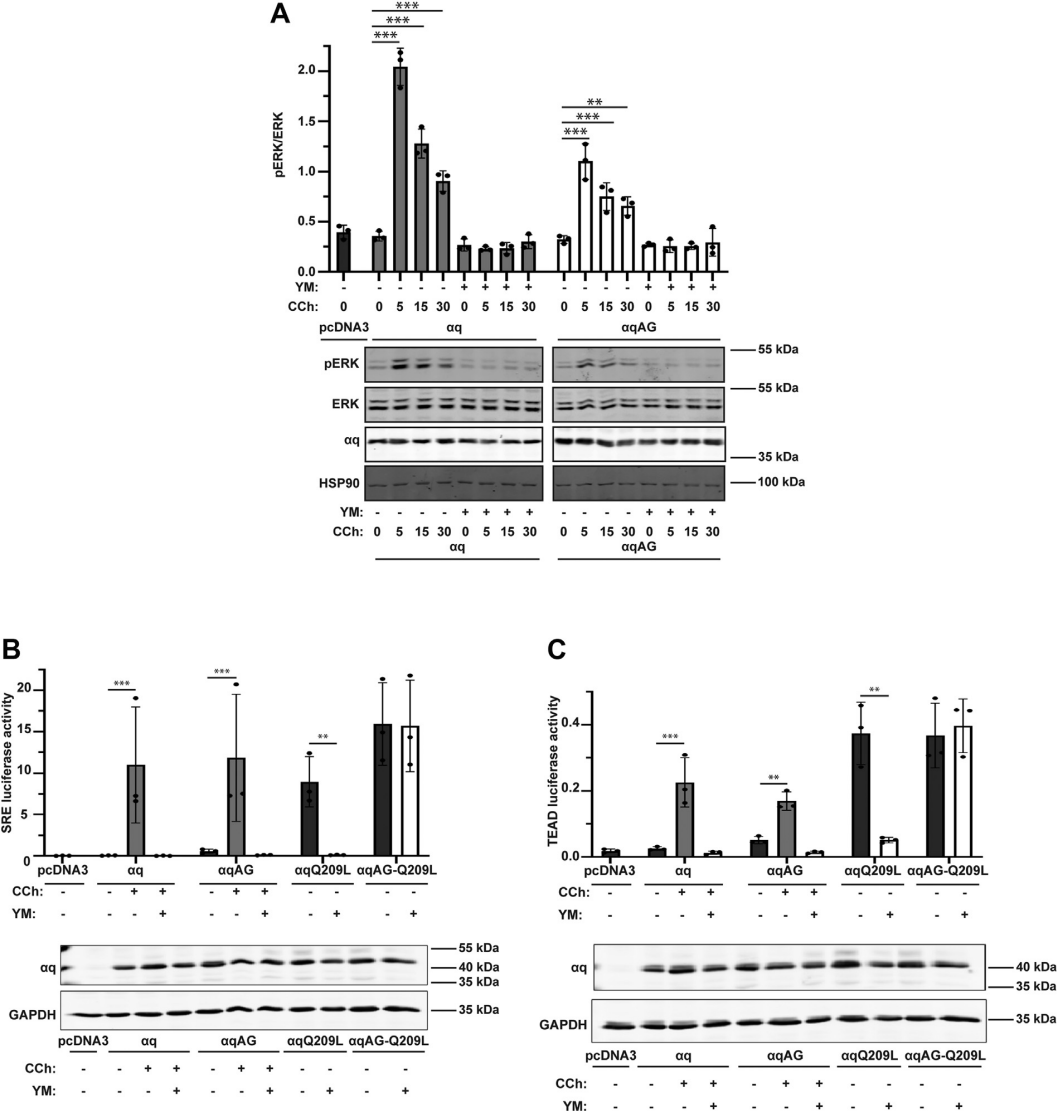
**GPCR-activated αqAG is sensitive to YM.***A*, HEK 293 q/11 K/O cells were transfected with αq or αqAG, then treated with 100 μM carbachol for 5, 15, or 30 min after 2 h pretreatment with 1 μM YM or DMSO vehicle control. Cell lysates were immunoblotted using antibodies for the indicated proteins and pERK/ERK signal intensities were quantified. Results are shown as mean ± SD (n = 3; ∗∗*p* <0.01; ∗∗∗*p* <0.001, two-way ANOVA, Dunnett’s multiple comparisons test). *B* and *C*, HEK 293 q/11 K/O cells were transfected with αq-pcDNA3, αqQ209L-pcDNA3, αqAG-Q209L, or pcDNA3 alone, along with muscarinic acetylcholine m3 receptor, renilla luciferase, and either pSRE luciferase (*B*) or 8xGTIIC (TEAD) luciferase (*C*). Cells were then treated with 100 μM carbachol overnight after a 1 h pretreatment with 1 μM YM or DMSO vehicle control. αq Q209L and αqAG-Q209L were treated with either 1 μM YM or DMSO vehicle control overnight. Luciferase assays were performed and quantitated as described previously. Cell lysates were immunoblotted using antibodies for the indicated proteins. Results are shown as mean ± SD (n = 3; ∗∗*p* <0.01; ∗∗∗*p* <0.001, two-way ANOVA, Šidák’s multiple comparisons test). DMSO, dimethyl sulfoxide; GPCE, G protein–coupled receptor.

We next wanted to determine if GPCR-dependent signaling by WT PM-restricted αq is also refractory to inhibition by YM. To test this, we transfected αq or αqAG into HEK 293 q/11 KO cells and then activated the endogenous muscarinic acetylcholine m3 receptor (m3AChR) with 100 μM carbachol in the presence or absence of pretreatment with YM for 2 h prior to carbachol treatment. Both αq and αqAG were able to activate the MAPK pathway within 5 min of carbachol treatment as assessed by immunoblotting for pERK (Fig. 6*A*). Importantly, treatment with YM abolished carbachol stimulation of the MAPK pathway for both αq or αqAG, indicating that signaling by GPCR-activated αqAG, in contrast to constitutive signaling by αqAG-Q209L, is fully inhibited by YM. To further assess the sensitivity of GPCR-activated αqAG to YM, we used the SRE luciferase reporter and TEAD luciferase reporter assays. Overnight treatment with carbachol resulted in robust stimulation of SRE luciferase and TEAD luciferase activity *via* either αq and αqAG (Fig. 6*B*), and similar to the results with pERK (Fig. 6*A*), both αq- and αqAG-mediated signaling was completely blocked by the addition of YM. Taken together, our results show that the surprising ability of PM-restricted αq to resist inhibition by YM is unique to the constitutively active Q209L mutants; GPCR-activated signaling by PM-restricted αq retains sensitivity to YM.

## Discussion

The studies presented here suggest a novel mechanism for inhibition of constitutively active αqQ209L by the depsipeptide YM-254890 (YM). It is well accepted that YM and the very similar FR900359 (FR) inhibit GPCR activation of αq by locking αq in the GDP-bound state (17, 21, 23). In addition to inhibiting classical GPCR activation of αq, recent work has shown that YM can inhibit signaling and cell proliferation driven by αqQ209L (18, 19, 20). However, constitutively active αqQ209L is assumed to exist in a GTP-bound active state due to its lack of GTPase activity, and thus an understanding of how YM could similarly trap constitutively active αqQ209L in an inactive state has remained a challenge. Results herein address this challenge by supporting an additional mechanism for YM inhibition of αqQ209L. The key findings are that (1) YM promotes subcellular redistribution of αqQ209L off of the PM and (2) YM fails to inhibit signaling by PM-restricted mutants of αqQ209L. Our results propose a model in which YM binding promotes the dissociation of αqQ209L from the PM, and the resulting decreased PM localization prevents αqQ209L signaling.

Our studies have revealed an unexpected subcellular redistribution of αqQ209L in response to YM treatment. Importantly, previous work on the trafficking of Gα subunits has led to a model in which Gα can reversibly shuttle between the PM and internal membranes in both constitutive and activation-dependent manners (29, 30, 36, 37, 38, 39). Although it is clear that G proteins can traffic to different locations in the cell, a detailed mechanistic understanding remains to be defined. The main insight into a mechanism is that changes in palmitoylation and depalmitoylation of Gα can play a key role in their trafficking (29, 40). In this regard, it was reported that αq fused to a photoconvertible fluorescent protein could rapidly shuttle between the PM and intracellular membranes and also that blocking palmitoylation of αq by treatment with 2-bromopalmitate or depletion of specific palmitoyl acyltransferases led to loss of PM localization of αq (29). Thus, an attractive model for our observation of YM-promoted redistribution of αqQ209L is that binding of YM by αqQ209L leads to changes in its palmitoylation status. However, in initial studies, we have not been able to demonstrate consistent changes in palmitoylation of αq or αqQ209L when cells are treated with YM. Moreover, our studies so far have not been able to conclusively determine the subcellular localization of αqQ209L upon translocation from the PM. After YM treatment, αqQ209L appears diffusely localized throughout the cell with no clear colocalization with intracellular organelles. Intriguingly, cellular fractionation experiments show a small shift of αqQ209L into a soluble, cytosolic fraction after YM treatment, consistent with a potential mechanism of depalmitoylation (Fig. S2). Future studies will be needed to understand the mechanism of this YM-promoted redistribution of αqQ209L; it is currently unclear whether YM binding by αqQ209L stimulates retrograde movement off of the PM or inhibits anterograde trafficking for return to the PM. To our knowledge, results reported herein are the first demonstration of a small molecule binding to a heterotrimeric G protein subunit that affects its subcellular localization. YM is likely to provide an important tool to help unravel trafficking mechanisms of Gα.

The most compelling findings in our studies were that simply adding additional membrane binding motifs to the N terminus of αqQ209L were sufficient to prevent YM from inhibiting signaling by these mutant αqQ209L. Palmitoylation at cysteines 9 and 10 is essential for PM localization and signaling of WT and constitutively active mutants of αq, and cycles of enzymatic palmitoylation and depalmitoylation can control the subcellular distribution of αq proteins. We reasoned that appending additional membrane binding motifs to αqQ209L would result in a stronger attachment to the PM, and indeed N-terminal fusion of a myristoylation + basic sequence from Src, a myristoylation + palmitoylation sequence from Lyn, or the simple AG mutation to introduce a site of myristoylation resulted in PM-restricted mutants of αqQ209L that were predominantly localized at the PM and remained at the PM upon YM treatment. Signaling by Src-αqQ209L, Lyn-αqQ209L, and αqAG-Q209L failed to be inhibited by YM. Moreover, we demonstrated that insensitivity to YM was maintained at different concentrations of YM and different expression levels of αqAG-Q209L (Fig. 4, *A* and *B*). A previous study reported an IC50 of 12 nM YM for inhibiting signaling by αqQ209L (41), consistent with the YM concentration curve in our studies (Fig. 4*B*). Nonetheless, concentrations of up to 5 μM YM still failed to inhibit αqAG-Q209L. Increasing expression of αqQ209L led to increased signaling, and YM fully inhibited signaling by αqQ209L at all levels of expression; however, YM was unable to inhibit αqAG-Q209L signaling at any level of expression (Fig. 4*A*). Taken together, the failure of YM to inhibit the PM-restricted forms of αqQ209L supports a model in which translocation off of the PM contributes to the mechanism of YM inhibition of αqQ209L.

The surprising resistance to YM of PM-restricted mutants was specific to the constitutively active αqQ209L. In our studies, both WT αq and WT αqAG efficiently and similarly coupled carbachol-stimulated endogenous m3AChR or expressed m3AchR to activation of ERK and stimulation of luciferase readouts, respectively. However, in these assays, signaling by both WT αq and αqAG were able to be completely inhibited by YM. This sensitivity to YM of GPCR-stimulated signaling by αqAG was in contrast to our demonstrations that signaling by αqAG-Q209L was insensitive to YM. These results are consistent with the proposal that there are key mechanistic differences in cells regarding how WT αq *versus* GTPase-deficient αq mutants are inhibited by YM.

A key question that we addressed was whether PM-restricted αqAG-Q209L is capable of binding YM. Importantly, although our experiments showed that signaling by PM-restricted αqAG-Q209L is insensitive to YM, our studies determined that YM is still able to bind PM-restricted αqAG-Q209L and promote conformational changes similar to those that occur upon YM binding by αqQ209L. Based on a crystal structure of YM bound to WT αq and biochemical studies with YM or the highly similar FR900359, the accepted mechanism of action is that YM binds to GDP-bound WT αq and thereby locks it in the inactive state (19, 21, 23). Moreover, cell-based studies have indicated that FR900359 binding can also promote a conformation in αqQ209L, consistent with it being in an inactive, GDP-bound form (18, 19). Our studies used pull-down experiments to show that αqQ209L displayed increased association with Gβγ and decreased association with RGS2, p63RhoGEF-DH/PHext, and PLCβ after treatment of cells with YM (Fig. 5), showing that YM can promote an inactive conformation of αqQ209L, consistent with similar reported experiments using FR900359 (19). Critically, αqAG-Q209L showed the same YM-dependent changes of interaction with these binding partners as did αqQ209L. Thus, the failure of YM to inhibit signaling by αqAG-Q209L cannot be ascribed to an inability of αqAG-Q209L to bind YM.

How then does signaling by PM-restricted αqQ209L mutants avoid inhibition by YM? Taken together, our results suggest a model in which YM-promoted subcellular redistribution of αqQ209L contributes to the mechanism of inhibition, in addition to the already recognized potential of YM to shift αq subunits into an inactive conformation. In this model, YM binding induces translocation of αqQ209L off of the PM; the redistributed αqQ209L is then unable to stimulate effector proteins and signaling pathways. This is supported by reports showing that αq and constitutively active mutants of αq completely lack signaling function when PM localization is disrupted (25, 26, 27, 28, 29). PM-restricted αqQ209L mutants, on the other hand, remain associated with the PM in the presence of YM and thus continue to signal, even if bound to YM. Furthermore, our results showing that YM promotes a similar conformation in both αqQ209L and αqAG-Q209L raises the possibility that YM binding to constitutively active αqQ209L and the apparent resulting inactivating conformational change, may not be sufficient for inhibition by YM. Because αqQ209L lacks GTPase activity, it is assumed that a substantial proportion of αqQ209L exist in the active, GTP-bound form in cells; however, it is also assumed that YM binds to an inactive, GDP-bound form of αqQ209L, and thus it remains to be fully understood how YM could rapidly lock enough αqQ209L in a GDP-bound form to fully inhibit signaling. It is worthwhile to note a recent study of constitutively active αsR201C (42). The authors demonstrated that GDP-bound αsR201C can exist in an active conformation capable of activating the effector adenylyl cyclase, contrary to assumptions that αsR201C would be inactive when bound to GDP. Thus, the results of Hu and Shokat raise the possibility that YM binding to αqQ209L and the resulting shift to a GDP form, may not be sufficient to generate an inactive αqQ209L; additional mechanisms, such as subcellular redistribution, may be necessary. Further support for our model is our demonstration that GPCR-stimulated signaling *via* WT αq or WT αqAG is efficiently inhibited by YM treatment. These WT αq are likely to be predominantly in the GDP-bound inactive form when YM is added to cells, allowing YM to rapidly lock them in an inactive form. Thus, additional mechanisms of YM action are likely not required for inhibition of WT αq, highlighting the uniqueness of constitutively active αqQ209L.

An additional model to explain the insensitivity of PM-restricted αqQ209L mutants is that the addition of N-terminal membrane-binding motifs affects the ability of αqQ209L to bind key regulatory proteins that may help mediate the inhibitory effect of YM on αqQ209L. For example, as of yet unidentified proteins may facilitate the inhibitory effect of YM by mediating relocalization of αqQ209L, but such proteins may not effectively interact with PM-restricted αqQ209L. Likewise, other proteins may prevent the inhibitory effect of YM by maintaining signaling function of PM-restricted αqQ209L. In this regard, a recent report argued that the proportion of αqQ209L already bound to the effector PLCβ would be refractory to inhibition by FR, an inhibitor almost identical to YM; because αqQ209L would not be susceptible to the GTPase accelerating protein (GAP) activity of PLCβ, the αqQ209L-PLCβ complex would be longer lived than an αq-PLCβ complex and consequently inaccessible to the inhibitor FR or YM (20). In this model, we can speculate that a PM-restricted αqQ209L, with tighter membrane binding than αqQ209L, may exist in a membrane-bound complex with PLCβ and other proteins that is more difficult to disrupt and thus less able to be inhibited by YM. However, in contrast to this idea, our immunoprecipitation studies failed to show any increase in association of αqAG-Q209L with PLCβ-3 compared to αqQ209L association with PLCβ-3 (Fig. 5*D*). Nonetheless, we cannot rule out that in the intact cell αqAG-Q209L, compared to αqQ209L, is more strongly associated with PLCβ-3 or other interacting proteins. Future studies to identify novel proteins that regulate inhibition of αqQ209L and structural studies to understand how YM and/or GDP binding affects αqQ209L are needed to more fully understand YM inhibition of constitutively active αq mutants.

In summary, our studies show that YM promotes a change in localization of αqQ209L from the PM to cytoplasm and that restricting αqQ209L to the PM results in a loss of inhibition by YM, suggesting that this change in localization is important for YM’s mechanism of action. The mutation of Q209L in αq or the highly identical α11, is the major oncogenic driver mutation in uveal melanoma. A deeper understanding of how the depsipeptide inhibitor YM inhibits αqQ209L will guide the development of new inhibitors and potentially reveal new therapeutic targets.

## Experimental procedures

### Reagents, antibodies, and plasmids

YM-254890 (catalog no.: # 257-00631) was purchased from Wako Chemicals USA Inc. DMSO (catalog no.: # BP231-1) was purchased from Fisher. HEK 293 cells and HEK293 αq/11 K/O cells were cultured in Dulbecco's modified Eagle's medium (Corning, catalog no.: # 10-017-CV) containing 10% fetal bovine serum (Gemini, catalog no.: 900-108) and 1× penicillin/streptomycin solution (Sigma, catalog no.: # P4333). All other cell culture plates and materials were from GenClone, Costar, or Fisher. Lipofectamine 2000 (catalog no.: # 11668-019), used for all transient transfections, was obtained from Invitrogen. Ponceau S (catalog no.: # P-3504) and carbachol were obtained from Sigma–Aldrich.

The following primary antibodies were used. The GAPDH (catalog no.: # 60004-1-Ig) and αq (catalog no.: #13927-1-AP) antibodies were purchased from ProteinTech. This αq antibody (catalog no.: #13927-1-AP) was used for all immunofluorescence microscopy experiments together with a GRK4-6, clone A16/17 (catalog no.: #05-466) antibody, which was obtained from Sigma–Aldrich. An additional αq antibody (catalog no.: # ab199533) was purchased from Abcam and was used for all immunoblotting experiments. The HSP90 antibody (catalog no.: # sc-7947) was obtained from Santa Cruz Biotechnology. The pERK1/2 (catalog no.: #9101S) and ERK1/2 (catalog no.: #4696S) antibodies were obtained from Cell Signaling Technologies. The anti-myc tag antibody, clone 9E1 (catalog no.: # 05-419) was obtained from Sigma–Aldrich. For immunofluorescence microscopy, the secondary goat anti-rabbit Alexa Fluor 488 (catalog no.: # A-11034) and goat antimouse Alexa Fluor 594 (catalog no.: # A-11032) conjugated secondary antibodies were obtained from Invitrogen. The secondary LI-COR antibodies, IRDye 800CW donkey antimouse IgG (H + L) (catalog no.: # 92532212), and IRDye 680RD goat anti-rabbit IgG (H + L) (catalog no.: #92568071) were purchased from LI-COR and were used to visualize protein on immunoblots. Alternatively for immunoblotting, horseradish peroxidase-conjugated secondary antibodies (catalog no.: # PR-W4011 and PR-W4021) were used from Promega.

HA-tagged αq, αqQ209L, αqAG, and αqAGQ209L in pcDNA3 were described previously (25, 26, 27). Nontagged human αq (catalog no.: # GNA0Q00000) and αqQ209L (catalog no.: # GNA0Q000C0) in pcDNA3.1 were received from the complementary DNA Resource Center. Src-αqQ209L and Lyn-αqQ209L in pcDNA3 were generated using synthetic DNA (GenScript) and subcloning into HA-tagged αqQ209L-pcDNA3 and resulted in coding sequences for amino acids 1 to 16 of Src and 1 to 11 of Lyn, respectively, followed by DNA coding for the linker Gly-Thr-Gly-Gly-Ser-Gly-Gly-Gly-Ser-Gly-Gly-Gly-Ser-Gly-Ala and then fusion to Thr2 of αqQ209L. pmCherry(N1)-GRK5, in which mCherry is fused to the C terminus of GRK5 was described previously (31), and the bacteria expression plasmid for GST-RGS2 has been described (43, 44). A bacteria expression plasmid for GST-p63RhoGEF-DH/PHext and pcDNA3.1-FLAG-PLCβ-3 were generously provided by Mikel Garcia-Marcos (Boston University).

### Cell lines

HEK 293 cells were received from ATCC. HEK 293 q/11 K/O cells have been described (17). HEK 293 cells stably expressing myc-His-tagged β1 and γ2 were described previously (26). The cells were maintained in HEK293 cell media plus 0.5 mg/ml G418. For generation of HEK 293 cells expressing tetracycline-induced αq and αqQ209L, HEK 293 cells stably expressing an integrated Flp Recombination Target (FRT) site and Tet repressor (TR) (Flp-In T-REx HEK 293 cells) were received from Diane Merry (Jefferson) and were cultured in HEK 293 media plus 15 μg/ml blasticidin and 100 μg/ml zeocin. HA-tagged αq and αqQ209L complementary DNA in a pcDNA3 vector backbone were cut using the restriction endonucleases Hind III and Apa I and ligated into the pcDNA5/FRT/TO expression vector, which contained a hybrid human cytomegalovirus (CMV)/TetO_2_ promotor for tetracycline-regulated expression of αq or αqQ209L, FRT site for Flp recombinase–mediated integration of the vector into the Flp-In T-REx HEK 293 cells, and a hygromycin resistance gene for selection. The HEK 293 cells were transfected with 2 μg of HA-tagged αq-pcDNA5/FRT/TO or αqQ209L-pcDNA5/FRT/TO plus 13 μg of POGG44 flip recombinase. HEK 293 cells with integrated αq or αqQ209L were selected by using HEK 293 media plus 15 μg/ml blasticidin and 100 μg/ml hygromycin and single colonies were expanded under blasticidin and hygromycin selection. αq and αqQ209L Flp-In T-REx HEK293 cell clones were validated by observation of equal expression across cells by immunofluorescence upon 100 nM tetracycline treatment and nuclear YAP and increased pERK upon tetracycline treatment in αqQ209L Flp-In T-REx HEK 293 cells.

### Immunofluorescence microscopy

Immunofluorescence microscopy experiments involved coexpression of αq or αqQ209L and GRK5-mCherry-N1, which localizes at the PM and does not interact with αq (31, 45). αq and αqQ209L Flp-In T-REx HEK 293 cells were initially transfected with GRK5-mCherry-N1 before tetracycline treatment then reseeded onto 6-well plates with coverslips 24 h after transfection. About 100 ng/ml tetracycline was then used to induce expression of αq and αqQ209L. The cells were then treated with DMSO or 1 μM YM in DMSO overnight or for 1 h prior to fixing. To detect localization of Src-αqQ209L or Lyn-αqQ209L HEK 293 q/11 K/O cells were seeded onto poly-L-lysine-coated coverslips and transfected with Src-αqQ209L or Lyn-αqQ209L plus GRK5-mCherry-N1. Forty-eight hours after transfection, the cells were treated with YM for 1 h then fixed. All cells were fixed with 3.7% formaldehyde in PBS for 15 min, then washed three times with PBS. The cells were then blocked and permeabilized by incubation with 2.5% milk and 1% Triton X-100 in tris-buffered saline (TBS) for 20 min. Coverslips were then incubated with an anti-rabbit αq antibody and antimouse GRK 4 to 6 antibody in 2.5% milk and 1% Triton X-100 in TBS for 1 h. The cells were then washed five times in 2.5% milk and 1% Triton X-100 in TBS then incubated with goat anti-rabbit Alexa Fluor 488 and goat antimouse Alexa Fluor 594 secondary antibodies in 1% Triton X-100 in TBS for 30 min. After five washes in 1% Triton X-100 in TBS, the cells were rinsed in distilled water and mounted onto glass slides with ProLong Diamond Anti-fade Mountant (Invitrogen, catalog no.: # P36970). Images were acquired on an Olympus IX83 microscope with a 60× oil immersion objective and an ORCA Fusion sCMOS camera (Hamamatsu) controlled by cellSense (Olympus software). Images were subjected to constrained iterative deconvolution to remove background fluorescence. Hundred tetracycline-induced αq and αqQ209L Flp-In HEK 293 cells under each treatment were counted and scored as either PM localized with little to no observable staining in the cytoplasm, PM, and cytoplasmic localization in which individual cells displayed varying degrees of a partial PM stain and observable cytoplasmic localization of αq or cytoplasmic in which αq was distributed throughout the cytoplasm but had no observable PM localization.

### Dual luciferase assay

HEK 293 αq/11 K/O cells were cotransfected with *Renilla* luciferase and either pSRE-luciferase or the synthetic TEAD reporter 8xGTIIC-luciferase along with the respective αq construct. 8xGTIIC-luciferase was received from Stefano Piccolo (Addgene plasmid # 34615) and pSRE-luciferase was described previously (46). For experiments using constitutively active mutants, the media was changed to serum-free media plus 1 μM YM or DMSO vehicle control 2 h after transfection, then lysed approximately 16 h after transfection. For experiments involving GPCR-activated αq, the cells were treated with 1 μM YM or DMSO vehicle control 2 h after transfection, treated with 100 μM carbachol or vehicle control 1 h after DMSO or YM treatment, and then lysed approximately 16 h after transfection. The cells were lysed in 1× passive lysis buffer and luciferase activity was detected using the Dual-Luciferase Reporter Assay System kit (Promega, catalog no.: # E1960) according to the manufacturer’s directions, using an opaque white 96-well plate and GloMax Explorer luminometer.

### βγ pull-down assay

The βγ pull-down assay was done as described in (26). Briefly, HEK 293 cells stably expressing β1γ2 were transiently transfected with HA-tagged αq-pcDNA3, αqQ209L-pcDNA3, αqAG-pcDNA3, αqAG-Q209L-pcDNA3, or pcDNA3 alone. Twenty-four hours after transfection, the cells were treated with 1 μM YM or DMSO vehicle control overnight. The cells were then washed in PBS and lysed in 500 μl of lysis buffer C (20 mM Hepes pH 7.5, 100 mM NaCl, 0.7% Triton X-100, 5 mM MgCl2, and 1 mM EDTA supplemented with the protease inhibitors 1 mM PMSF, 2 μg/ml leupeptin, and 2 μg/ml aprotinin, and 0.1× cOmplete mini protease inhibitor cocktail). The lysates were incubated for 1 h on ice then centrifuged at 13,000 rpm (10,000*g*) for 3 min to pellet the nuclei and insoluble material. Forty microliters of the supernatant was reserved for the input, and 20 μl of Ni-NTA magnetic beads (New England Biolabs, catalog no.: #S1423S) were added to a new tube with the remaining supernatant. The tubes were rotated e-o-e for 2 h at 4 °C. The beads were then pelleted by centrifugation for 1 min, then the supernatant was aspirated while the beads were pelleted on a magnetic rack. The beads were washed three times in lysis buffer C to remove nonspecific proteins. Fifty microliters of elution buffer (0.25 M imidazole in lysis buffer C) was added to each sample to elute poly-His β1 and any associated proteins. SDS-PAGE sample buffer with 1% β-mercaptoethanol were added to the Ni-NTA beads and the whole cell lysates. The pull down and input were separated by SDS-PAGE, transferred onto nitrocellulose membrane, and probed with an αq (Abcam, catalog no.: # ab199533) or an anti-myc monoclonal antibody (9E10) to detect β1 or HSP90 antibody as a loading control. The bands were visualized by chemiluminescence and subjected to densitometry. Each sample was normalized to its respective DMSO treatment.

### GST-RGS2 and GST-p63RhoGEF-DH/PHext pull-down assays

The GST-RGS2 and GST-p63RhoGEF-DH/PHext pull-down assays was performed as described in (35, 43). Briefly, GST-RGS2 and GST-p63RhoGEF-DH/PHext was expressed in BL-21 cells and BL-21(DE3) cells, respectively. GST-RGS2 expression was induced with 0.5 mM IPTG for 3 h at 25 °C and GST-p63RhoGEF-DH/PHext was induced with 1 mM IPTG overnight at 18 °C. The GST fusion proteins were then purified using GSH-Sepharose 4B beads (GE Healthcare, catalog no.: # 17-0765-01) as described (35, 44). HEK 293 q/11 K/O cells were transfected with pcDNA3, HA-tagged αq-pcDNA3 or αqQ209L-pcDNA3, or αqAG-Q209L-pcDNA3. Twenty-four hours after transfection, the cells were treated with 1 μM YM or DMSO vehicle control overnight. The cells were then washed with cold PBS and lysed in 300 μl of lysis buffer (20 mM Tris–HCl, pH 7.4, 1 mM EDTA, 1 mM DTT, 100 nM NaCl, 5 mM MgCl2, 0.7% Triton X-100, 1 mM PMSF, and 5 μg/ml leupeptin and aprotinin). The cells were lysed on ice for 1 h, then centrifuged for 3 min at full speed to pellet the nuclei and insoluble material. About 50 μl of the supernatant was reserved for the input and the remaining supernatant was added to a new tube with 8 μg of GST-RGS2 or GST-p63RhoGEF-DH/PHext prebound to GSH Sepharose beads and rotated e-o-e at 4 °C for 1 h. After incubation with GST-RGS2 or GST-p63RhoGEF-DH/PHext, the samples were pelleted at 13,000 rpm (10,000*g*) for 1 min, the flow through was removed and the beads were washed three times in lysis buffer. The proteins were eluted from the beads in 50 μl of SDS sample buffer plus 1% β-mercaptoethanol and the protein was separated by SDS-PAGE and transferred onto nitrocellulose membrane then probed with an antibody detecting αq (Abcam) or HSP90 as a loading control. GST-RGS2 and GST-p63RhoGEF-DH/PHext were detected by staining the membrane with Ponceau S. The bands were visualized using LI-COR secondary antibodies and subjected to densitometry. Each sample was normalized to its respective DMSO treatment.

### PLCβ-3 immunoprecipitation

Experiments were performed similarly to a recent report (35). HEK 293 q/11 KO cells were transiently transfected with FLAG-tagged PLCβ-3 and αqQ209L-pcDNA3 or αqAG-Q209L-pcDNA3. FLAG-tagged PLCβ-3 alone and pcDNA3 alone were transfected as positive and negative controls, respectively. Three hours after transfection, media was changed and respective plates were treated with 1 μM YM overnight. The cells were washed with cold PBS and lysed in 500 μl of lysis buffer C (20 mM Hepes pH 7.5, 100 mM NaCl, 0.7% Triton X-100, 5 mM MgCl2, and 1 mM EDTA supplemented with 1 mM DTT, 1 mM PMSF, 2 μg/ml leupeptin, and 2 μg/ml aprotinin, and 0.1× cOmplete mini protease inhibitor cocktail). Lysates were incubated for 30 min on ice and then centrifuged at 13,000 rpm for 10 min. Fifty microliters of the supernatant was reserved for the input, while the remaining supernatant was added to a new tube containing 40 μl of EZview Red ANTI-FLAG M2 Affinity Gel bead slurry (Sigma––Aldrich, catalog no.: # F2426). Lysates and beads were rotated e-o-e for 2 h at 4 °C. The beads were then pelleted by centrifugation for 1 min. Supernatant was removed, and the beads were washed three times with lysis buffer C. Protein was eluted from the beads in 60 μl of 1× SDS sample buffer plus 1% β-mercaptoethanol. Pull-down and input samples were run on 10% SDS-PAGE gels, transferred onto nitrocellulose membrane, and probed with antibodies detecting PLCβ-3 (Sigma–Aldrich ANTI-FLAG M2, catalog no.: # F1804), αq (ProteinTech, catalog no.: #13927-1-AP), and GAPDH as a loading control. The bands were visualized using LI-COR secondary antibodies and subjected to densitometry. Coimmunoprecipitated αq was normalized to the amount of PLCβ-3 detected in the immunoprecipitate.

### pERK/ERK analysis

Protein lysates were run on 10% SDS-PAGE gels then transferred onto nitrocellulose membranes. The membranes were then probed for with a rabbit polyclonal pERK antibody (catalog no.: # 9101S) or a mouse monoclonal ERK antibody (catalog no.: #4696S). A duplicate membrane was probed with an antibody detecting αq (Abcam) or GAPDH. LI-COR secondary antibodies were used to visualize the protein bands. The pERK and ERK bands were subjected to densitometry and the signal obtained from the pERK bands were divided by the signal detected from ERK. All signals were normalized to 0 h YM treatment to normalize between experiments.

### Western blotting and quantification

All protein lysates were run on 10% SDS-PAGE gels and transferred onto nitrocellulose, blocked in 2.5% bovine serum albumin (BSA) or 5% milk in TBS plus 0.05% Tween (TBS-Tween), incubated with primary antibodies overnight in 2.5% BSA or 5% milk in TBS-Tween, washed three times in TBS-Tween, incubated with secondary antibodies in 2.5% BSA or 5% milk in TBS-Tween for 1 h, and washed three times in TBS-Tween. Membranes blotted with LI-COR secondary antibodies were then washed once in PBS then visualized on a LI-COR Odyssey. Membranes blotted with horseradish peroxidase–conjugated secondary antibodies were incubated with SuperSignal West Dura Extended Duration Substrate (catalog no.: # 34075) from Thermo Scientific Dura then imaged on an Amersham Imager 680 (GE Healthcare).

### Statistical analysis

All figures were analyzed using GraphPad Prism (GraphPad Software Inc). A two-way ANOVA followed by multiple comparison’s testing was used to calculate significance. Multiple comparison testing was performed using Tukey's test when all groups were compared with all other groups, and a Dunnett's test was used when all groups were compared with a single control group. Šidák’s test was performed when DMSO *versus* YM treatments were compared as opposed to Tukey's test, which was used to compare all groups with all other groups. The Fisher's least significant differences test was used for the cell count comparison in Figures 1*B* and S1 and the cell fractionation in Fig. S2 to compare all groups without correcting for multiple comparisons. *t* tests were used in Figure 5 to compare to DMSO treatments set at 1. In all experiments, error bars indicate mean ± SD with significant differences indicated as ∗, *p* < 0.05; ∗∗*p* < 0.01; ∗∗∗*p* < 0.001.

## Data availability

All data is contained within the paper.

## Supporting information

This article contains supporting information.

## Conflict of interest

The authors declare that they have no conflicts of interest with the contents of this article.
