# Supplementary material for: Enhanced membrane binding of oncogenic G protein αqQ209L confers resistance to inhibitor YM-254890
Source: J Biol Chem. 2022 Sep 27;298(11):102538. doi: 10.1016/j.jbc.2022.102538 (PMC9626947; doi:10.1016/j.jbc.2022.102538)
Supplement: Figure S1 [file mmc1.pdf]

A

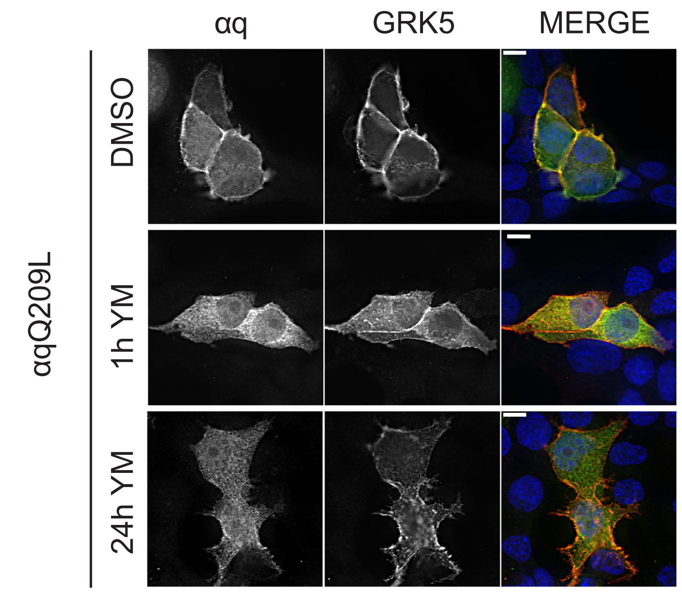

αq
GRK5
MERGE
DMSO
1h YM
24h YM
αqQ209L

B

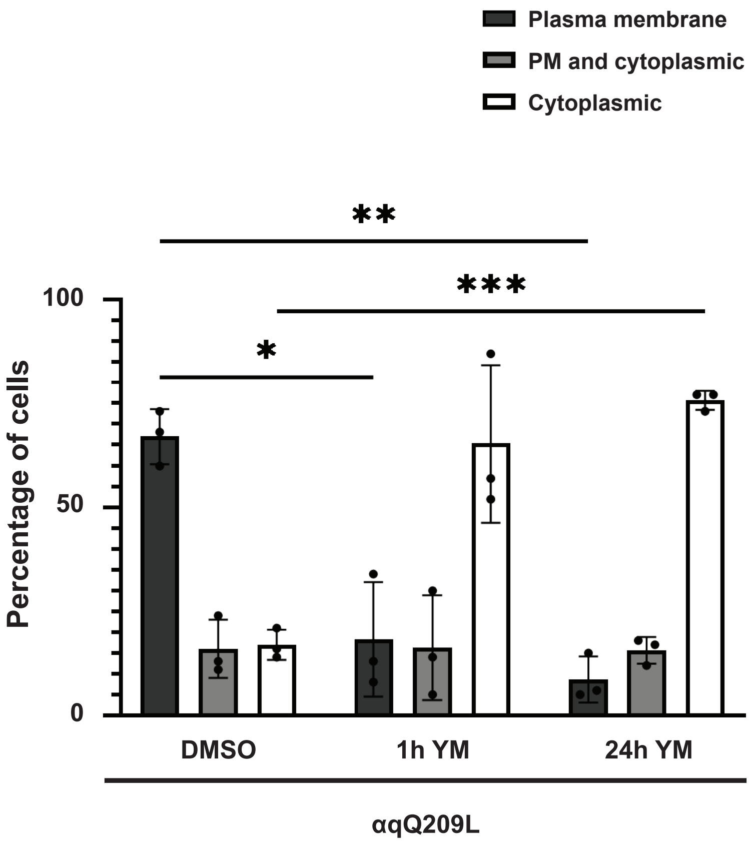

Plasma membrane
PM and cytoplasmic
Cytoplasmic
**
***
*
Percentage of cells
100
50
0
DMSO
1h YM
24h YM
αqQ209L

Supplementary Figure S1: **YM promotes dissociation of αqQ209L from the PM in HeLa cells.** (A) HeLa cells were co-transfected with αqQ209L and GRK5-mCherry expression vectors. cells were treated with DMSO or 1 μM YM for 1h or overnight. αq, αqQ209L and GRK5 were visualized by immunofluorescence microscopy, as described for Figure 1. Scale bars, 10 μM. (B) The localization of αqQ209L in 100 cells in each of n=3 experiments were scored as either PM-localized with little to no observable staining in the cytoplasm, PM and cytoplasmic localization in which individual cells displayed varying degrees of a partial PM stain and observable cytoplasmic localization of αq, or cytoplasmic in which αq was distributed throughout the cytoplasm, but had no observable PM localization. Results are shown as mean ± S.D. (n=3; experiments *, p <0.05, two-way ANOVA, Fisher's Least Significant Differences test).
