# Supplementary material for: Enhanced membrane binding of oncogenic G protein αqQ209L confers resistance to inhibitor YM-254890
Source: J Biol Chem. 2022 Sep 27;298(11):102538. doi: 10.1016/j.jbc.2022.102538 (PMC9626947; doi:10.1016/j.jbc.2022.102538)
Supplement: Figure S2 [file mmc2.pdf]

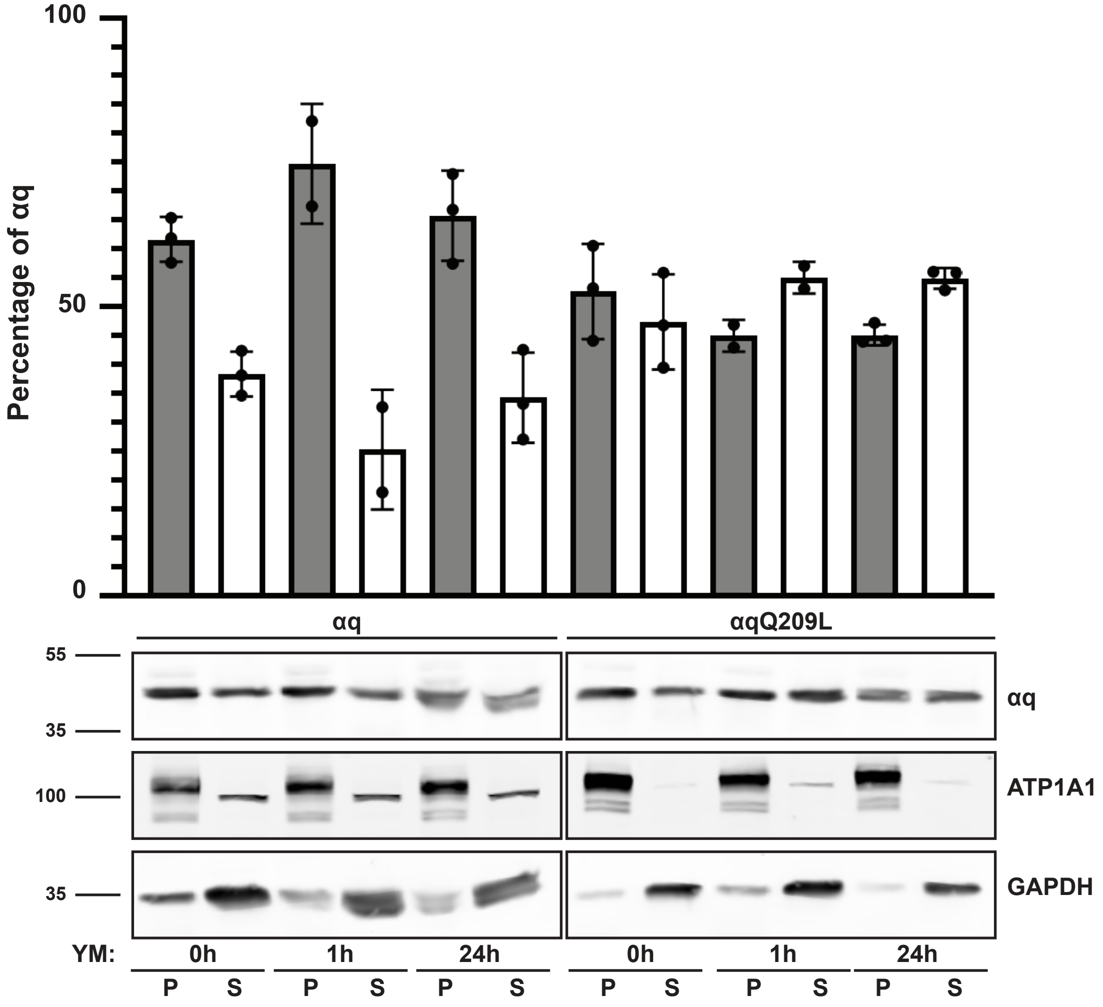
100
50
0
Percentage of αq
αq
αqQ209L
55
35
100
35
αq
ATP1A1
GAPDH
YM:
0h
1h
24h
0h
1h
24h
P S P S P S P S P S P S

Supplementary Figure S2: **Cell fractionation of αqQ209L -/+ YM.** αq and αqQ209L expression was induced with tetracycline in Flp-In HEK 293 cells, and cells were then treated with DMSO or 1 µM YM for 1h or overnight. Next, cells were washed in ice-cold PBS and lysed in hypotonic lysis buffer (50 mM Tris-HCl, pH 8, 2.5 mM MgCl2, 1 mM EDTA, 1 mM dithiothreitol + protease inhibitors) by passage through a 27-gauge needle 10 times. Lysed cells were centrifuged at 400×*g* for 5 min to remove nuclei and debris. The supernatant was centrifuged at 150,000×*g* for 20 min at 4 °C. The high-speed supernatant was designated the soluble (S) fraction. The high-speed pellet was solubilized in SDS-PAGE sample buffer and designated the particulate (P) fraction. Fractions were immunoblotted with antibodies against αq, Na/K ATPase (ATP1A1, membrane control) or GAPDH (cytosol control). Results are shown as mean ± S.D. (n=3, except for n=2 for 1 h YM).
